# Migraine Is More Than Just Headache: Is the Link to Chronic Fatigue and Mood Disorders Simply Due to Shared Biological Systems?

**DOI:** 10.3389/fnhum.2021.646692

**Published:** 2021-06-03

**Authors:** Nazia Karsan, Peter J. Goadsby

**Affiliations:** ^1^Headache Group, Wolfson Centre for Age-Related Diseases, Division of Neuroscience, Institute of Psychiatry, Psychology and Neuroscience, King’s College London, London, United Kingdom; ^2^NIHR-Wellcome Trust King’s Clinical Research Facility, SLaM Biomedical Research Centre, King’s College Hospital, London, United Kingdom; ^3^Department of Neurology, University of California, Los Angeles, Los Angeles, CA, United States

**Keywords:** migraine, mood, cognition, fatigue, sleep

## Abstract

Migraine is a symptomatically heterogeneous condition, of which headache is just one manifestation. Migraine is a disorder of altered sensory thresholding, with hypersensitivity among sufferers to sensory input. Advances in functional neuroimaging have highlighted that several brain areas are involved even prior to pain onset. Clinically, patients can experience symptoms hours to days prior to migraine pain, which can warn of impending headache. These symptoms can include mood and cognitive change, fatigue, and neck discomfort. Some epidemiological studies have suggested that migraine is associated in a bidirectional fashion with other disorders, such as mood disorders and chronic fatigue, as well as with other pain conditions such as fibromyalgia. This review will focus on the literature surrounding alterations in fatigue, mood, and cognition in particular, in association with migraine, and the suggested links to disorders such as chronic fatigue syndrome and depression. We hypothesize that migraine should be considered a neural disorder of brain function, in which alterations in aminergic networks integrating the limbic system with the sensory and homeostatic systems occur early and persist after headache resolution and perhaps interictally. The associations with some of these other disorders may allude to the inherent sensory sensitivity of the migraine brain and shared neurobiology and neurotransmitter systems rather than true co-morbidity.

## Introduction

Migraine is much more than a disorder of pain and involves symptomatic heterogeneity with a constellation of painful and painless symptoms, which can occur before, during, and after headache (Karsan and Goadsby, 2018). These symptoms can include mood and cognitive change and fatigue and disorders of arousal (Karsan et al., 2020b). Recognition of these symptoms being associated with the migraine attack by both patients and their physicians, particularly when they occur in the absence of headache, is variable and is likely to have increased with time (Bose et al., 2018; Karsan et al., 2018), not owing to increased prevalence but to increased understanding of the biology of migraine as a neural disorder of sensory processing, and therefore appreciation of these features of the attack (Goadsby et al., 2017).

Migraine is common, and while the global prevalence is around one in seven people (GBD 2016 Headache Collaborators, 2018), it is estimated that the lifetime consultation rate for headache in the United States is 79.8%, suggesting that, overall, migraine biology is more common than the rate of diagnosis suggests (Lipton et al., 2018). Epidemiological studies have suggested that migraine is associated with other systemic conditions such as depression (Bruti et al., 2012; Ligthart et al., 2013; Yang et al., 2016; Amoozegar, 2017; Peres et al., 2017; Amiri et al., 2019; Zhang et al., 2019), anxiety (Wacogne et al., 2003; Seng et al., 2017), irritable bowel syndrome (Cole et al., 2006; Cady et al., 2012; Chang and Lu, 2013; Lau et al., 2014; van Hemert et al., 2014; Cámara-Lemarroy et al., 2016; Le Gal et al., 2016; Perveen et al., 2016; Doulberis et al., 2017; Grassini and Nordin, 2017; Wu et al., 2017; Arzani et al., 2020), fibromyalgia (Nicolodi and Sicuteri, 1996; Peres et al., 2001; Peres, 2003; Marcus et al., 2005; Ifergane et al., 2006; Vij et al., 2015; Cho et al., 2017; Do et al., 2018; Whealy et al., 2018; Penn et al., 2019), sleep disorders (Cevoli et al., 2012; Engstrøm et al., 2013; Kim et al., 2017; Rains, 2018; Buse et al., 2019; Ferini-Strambi et al., 2019; Bertisch et al., 2020), and chronic fatigue (Peres et al., 2002; Lucchesi et al., 2016; Seo and Park, 2018), as well as cognitive disorders (Gil-Gouveia and Martins, 2017, 2019; Vuralli et al., 2018; Lo Buono et al., 2019). Many reasons have been postulated for these associations, including comorbidities, cause and effect, and shared pathophysiological mechanisms.

Human functional imaging studies have alluded to brain areas, which may be involved before (Maniyar et al., 2014; Schulte and May, 2016; Meylakh et al., 2018; Karsan et al., 2020a), during (Weiller et al., 1995; Bahra et al., 2001; Afridi M. et al., 2005; Afridi S. K. et al., 2005; Denuelle et al., 2007; Amin et al., 2016; Coppola et al., 2016a, 2018; Hougaard et al., 2017), and after migraine pain (Schulte and May, 2016; Marciszewski et al., 2018). The objective alterations in brain function in brain areas outside of the pain network, starting hours to days before headache onset, and persisting after headache resolution, associated with clinical symptoms during this time, indicate that each individual attack involves widespread brain dysfunction, within networks encompassing various neurotransmitter systems. These areas have been suggested to functionally correlate with the clinical symptoms experienced at each stage (Karsan and Goadsby, 2020). Brain regions that have been implicated include areas of the limbic pathway, hypothalamic and thalamic areas, and more typical regions within the pain network such as periaqueductal gray, amygdala, dorsolateral pons, and rostroventral medulla (Weiller et al., 1995; Bahra et al., 2001; Afridi M. et al., 2005; Afridi S. K. et al., 2005; Denuelle et al., 2007; Maniyar et al., 2014; Schulte and May, 2016). These brain areas overlap with those thought to be affected in mood disorders (Anand and Shekhar, 2003; Deckersbach et al., 2006; Savitz and Drevets, 2009; Price and Drevets, 2010; Scharinger et al., 2010; Myers-Schulz and Koenigs, 2012; Baker et al., 2019; Nugent et al., 2019; Stickel et al., 2019), as well as cognitive disorders (Devous, 2002; Herholz et al., 2007; Woodard and Sugarman, 2012; Roy et al., 2016; Bayer, 2018; Jalilianhasanpour et al., 2019) and disorders of arousal (Nofzinger, 2005a,b, 2008; Chee and Chuah, 2008; Desseilles et al., 2008; Dijk, 2012; Chee, 2013; Elvsåshagen et al., 2019). In addition, these different disorders are likely to be connected to and affected by each other; for example mood and cognitive disorders are often linked, in that low mood worsens cognition.

The paucity of objective measures used in routine clinical practice to quantify such patient complaints among migraineurs and the lack of change on any clinical investigations, such as structural brain imaging to account for these complaints, often lead to the mislabel of these as being psychosomatic in nature or attributed to the possible migraine-related worsening of coexistent mood and fatigue problems. While it is feasible that chronic pain of any kind could predispose to mood and fatigue issues, migraine has distinct features that differentiate it from other episodic and chronic pain conditions, in that there seems to be a somewhat distinct brain signature of the acute attack, which involves regions outside of the pain matrix within the brain, and seems to strongly involve activation of limbic pathways even prior to pain onset (Maniyar et al., 2014). Intermittent or continuous presence of similar symptomatology in those with more frequent attacks, or indeed chronic pain, is possible and, rather than suggesting the presence of three different disorders, which may be managed under different specialties, is perhaps more of an indicator of dynamic and perhaps long-term altered brain network dysfunction in migraine.

This review will focus on the literature supporting associations between migraine and some of these other conditions, as well as literature regarding brain areas and systems, and we therefore hypothesize that shared biological mechanisms between common conditions, rather than true comorbidity, may be the reason for these associations. In addition, the fact that migraine is a threshold disease with a hypersensitivity to sensory input is likely to contribute. This theory suggests that, in some cases, treating the migraine may have benefits on some of the other symptoms, although incorporation of these other non-headache symptoms into clinical trials and understanding of their effects on migraine-related disability and function are required to allow systematic evaluation of treatment effects going forward.

## Non-painful Symptoms in Migraine (Ictally During Any Phase or Interictally)

It has been alluded to as far back at the 19th century by Gowers (1899) that migraine involved prominent fatigue and lethargy. Despite this recognition, the full phenotype of symptoms that can be associated with migraine excluding headache and aura has been enhanced over the years, and it is now clear that symptoms can start hours to day prior to pain onset and in some individuals warn of impending headache (premonitory symptoms) (Giffin et al., 2003) and can persist during pain and following pain resolution (postdrome symptoms) (Giffin et al., 2016). The duration of time that any of these symptoms are present marks the entire duration of a single attack, which can be significantly longer than the canonical upper limit of 72 h, highlighted in the International Classification for Headache Disorders [Headache Classification Committee of the International Headache Society (IHS), 2018], thus prolonging the morbidity associated with each attack and altering function before, during, and after headache.

The premonitory phase has been studied in some detail in the literature over the years, in both adults and children, with a variety of study designs and patient populations (Blau, 1980; Drummond and Lance, 1984; Waelkens, 1985; Rasmussen and Olesen, 1992; Russell et al., 1996; Giffin et al., 2003; Kelman, 2004; Quintela et al., 2006; Schoonman et al., 2006; Cuvellier et al., 2009; Guo et al., 2016; Karsan et al., 2016, 2020b; Laurell et al., 2016; Jacobs and Pakalnis, 2019; Onderwater et al., 2020; Wang et al., 2021). While some studies have looked at prevalence and the ability of these symptoms to predict impending headache, others have focused on phenotype. Across four decades of studies, the similarities in phenotype reported when looking at the most common symptoms are remarkably consistent irrespective of patient population and study design. The most common symptoms reported during this time in both adults and children and adolescents are tiredness, yawning, and mood change.

In comparison, the postdrome phase is relatively much less studied. However, again over the years, a few studies have shown a phenotype dominated by fatigue and cognitive change after headache resolution, along with a number of other symptoms (Blau, 1982; Kelman, 2006; Ng-Mak et al., 2011; Bose et al., 2016; Giffin et al., 2016; Mamouri et al., 2018). These symptoms often impair return to normal function following headache resolution, sometimes up to days later. Studies suggest that these symptoms may be more common than premonitory symptoms (reported prevalences 7–88% for premonitory symptoms and 68–91% for postdrome symptoms).

It is therefore clear that from before the headache starts, through to even days after its resolution, migraine can be associated with dominant fatigue, mood, and cognitive change, among other symptoms. Ictal studies during headache have been more difficult to conduct, and perhaps these affective symptoms become less noticeable in the presence of pain, but there remains a suggestion that these alterations are also present during headache (Gil-Gouveia and Martins, 2017; Barbanti et al., 2020). Certainly, interictal alterations in cognitive function (Mulder et al., 1999; Martins et al., 2012), mood (Minen et al., 2016; Peres et al., 2017), and fatigue or arousal (Seo and Park, 2018) have been reported in migraine relative to healthy controls, suggesting that the inherent sensory sensitivity of the migraine brain may oscillate with attacks but may not be truly normal at baseline.

## Mood and Migraine

A study of 36,000 participants in a population-based Canadian study showed that major depression, bipolar disorder, panic disorder, and social phobia were all at least twice as prevalent in migraine subjects, and these findings were independent of demographic and socioeconomic variables (Jette et al., 2008). Similarly, anxiety is also common, with a cumulative lifetime incidence among migraineurs of 50% (Minen et al., 2016). Studies have estimated that depression is 2–2.5 times more likely to occur among migraineurs than healthy controls (Lipton et al., 2000; Breslau et al., 2003; Jette et al., 2008), and concomitant depression is present in approximately 40% of migraineurs (Lipton et al., 2000). This relationship seems to be bidirectional, with one disorder increasing the risk of the other (Breslau et al., 1994).

The risks of depression and anxiety in migraine are unsurprisingly related to headache burden, with one study showing a linear relationship between headache frequency and the odds ratio of depression or anxiety (Zwart et al., 2003) and another showing increased odds ratios for both depression and anxiety in chronic migraine compared to episodic migraine (Adams et al., 2015).

## Fatigue and Arousal in Migraine

Fatigue is a vague and multifactorial symptom, which is clearly influenced by sleep and arousal, systemic health, mood, and other exogenous factors including medications. While sleepiness and fatigue are different, there is clearly an interaction between them and a general difficulty among patients distinguishing the two. To our knowledge, the two separate issues or the distinction between them has not been studied in migraine studies. While it has been historically recognized that fatigue is a dominant feature of migraine (Gowers, 1899), more recently, associations with sleep disorders (Kelman and Rains, 2005; Nesbitt et al., 2014; Lucchesi et al., 2016; Rains, 2018; Buse et al., 2019) and shared physiological mechanisms with sleep pathways have been recognized (Holland and Goadsby, 2007; Holland, 2014, 2017; Vila-Pueyo et al., 2019). In addition, sleep disruption (oversleeping or undersleeping) is a commonly reported migraine trigger among sufferers (Pellegrino et al., 2018).

In general, it is estimated that approximately 60% of migraineurs report pathologic fatigue, based on several questionnaires including the Karolinska Sleepiness Scale, the Insomnia Severity Index, and the Fatigue Severity Scale (Seo and Park, 2018). Interestingly, emerging work using the novel CGRP pathway antibodies has suggested a beneficial impact on symptoms including fatigue and concentration on non-headache days and therefore improved function (VanderPluym et al., 2018).

At least a half of headache sufferers in one large study reported sleep disturbance (Kelman and Rains, 2005). A Norwegian-based population study found that migraineurs were three times more likely to have an Epworth Sleepiness Scale score of ≥10 compared to those without headache in nearly 300 subjects sampled and that migraineurs were five times more likely to have a high Karolinska Sleepiness Scale score compared to those without headache (Hagen et al., 2018).

## Shared Neuroanatomy and Neurotransmitter Systems

**FIGURE 1:**
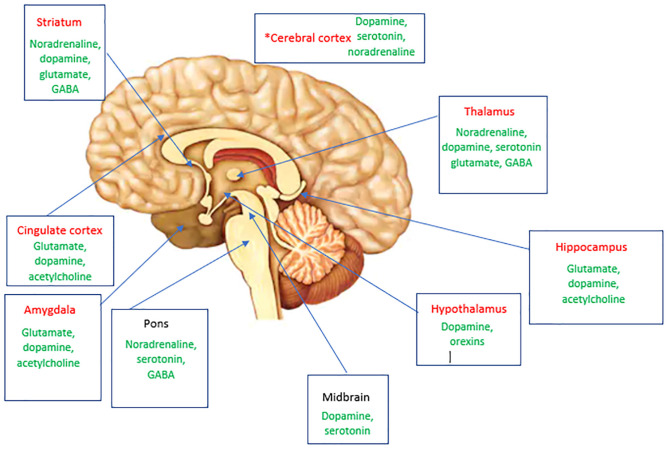
A summary of brain areas likely to be involved in mediating both pain and mood and arousal complaints in migraine via structural and functional connections. Thalamocortical pathways that modulate sensory and cognitive processing are also involved. Neurotransmitters implicated in these areas in migraine are shown in green. *Specific cortical areas implicated include dorsolateral prefrontal cortex, orbitofrontal cortex, anterior cingulate cortex, and the primary somatosensory cortex. Areas highlighted in red are likely to also have a role with their associated neurotransmitters in fatigue and mood.

We here propose some brain areas and neurotransmitter systems that may be shared in migraine and disorders of mood and fatigue (Figure 1). These brain areas and their functional and structural brain connections and corresponding neurotransmitter networks are likely involved in shared neurobiology between these disorders.

### Anterior Cingulate, Orbitofrontal, and Dorsolateral Prefrontal Cortex, as Well as Other Limbic Areas

The involvement of limbic areas on functional brain imaging in pain states is thought to be related to the higher processing of nociceptive input, cumulating the sensory, cognitive, and affective components of pain (Derbyshire et al., 1997; Tracey, 2008). The involvement of such areas prior to pain onset in migraine has been more recently shown (Maniyar et al., 2014) and suggests that this is not an affective or cognitive consequence of pain and is probably responsible for mediating attentional, mood, and cognitive deficits early in the attack. Some of these areas are part of the default mode network (DMN), which has been implicated in the sensory integration, cognitive, and attentional aspects of pain in migraine (Tessitore et al., 2013).

The anterior cingulate (ACC) is an area in the ventromedial frontal cortex often divided into anterior and posterior subregions anatomically, or affective and cognitive regions functionally (Devinsky et al., 1995). Historically, bilateral cingulotomy in psychiatric practice has been used to help treat severe treatment-resistant obsessive–compulsive disorder (OCD), chronic pain, depression, and substance abuse, and the success of this surgery (success rates of between 30 and 80% have been reported) has raised the likely role of the ACC in emotional behavior modulation (Gasquoine, 2013). Neuropsychological follow-up of patients exposed to this surgery has suggested a role of the ACC in cognition and executive functioning. Supportive functional neuroimaging findings have shown abnormal ACC activation or its involvement in OCD (Menzies et al., 2008), chronic pain (Peyron et al., 2000), and other conditions, including addictions. Other frontal cortical areas have also been implicated in these conditions, including the orbitofrontal cortex in OCD (Meunier et al., 2012; Weygandt et al., 2012) and cocaine addiction (Risinger et al., 2005).

In various pain states, involvement of the ACC on functional imaging studies is almost constantly observed (Watanabe et al., 2018; Shackman et al., 2011; Silvestrini et al., 2020), with a suggested role in affective and attentional responses to pain and in the selection of the response to pain. Additional areas of prefrontal cortex are thought to represent attentional and memory networks, which are also activated by noxious stimulation (Peyron et al., 2000). Support for prefrontal cortex involvement in pain states comes from functional neuroimaging evidence of the involvement of this brain area in acute pain in both clinical and experimental pain conditions (Apkarian et al., 2005). Even interictally in the absence of pain, migraineurs seem to display altered cerebral processing of negative and sensory stimuli, with increased activity on functional brain imaging in posterior cingulate cortex (PCC), caudate, amygdala, and thalamus (Wilcox et al., 2016), thereby implicating dysfunctional limbic networks in migraine, even in the absence of pain.

There have been studies demonstrating brain metabolite differences in the ACC of migraineurs compared to healthy controls using magnetic resonance spectroscopy, suggesting altered neurochemistry in this region in migraineurs. It is postulated that this difference may contribute to neuronal hyperexcitability in migraine (Becerra et al., 2016). Another study has also suggested orbitofrontal cortex hypofunction in migraine, in the context of medication overuse headache, using ^18^F-FDG positron emission tomography imaging. It was demonstrated that, although most of the pain matrix areas recovered to almost normal metabolism following medication withdrawal from hypometabolism during analgesic overuse, the orbitofrontal cortex remained hypoactive (Fumal et al., 2006).

Connectivity studies have demonstrated altered functional connectivity between these regions of cingulate and frontal cortex and other areas of interest in migraine. Russo et al. (2012); Tessitore et al. (2015), Yu et al. (2012), and Xue et al. (2013) have shown that migraineurs with and without aura in the resting state display reduced connectivity within regions of the frontoparietal network, including the middle frontal gyrus and ACC and areas of the DMN such as ACC, prefrontal cortex, and orbitofrontal cortex, relative to healthy controls. Other functional imaging studies have also shown that some of these regions may have altered metabolism in migraineurs (Kim et al., 2010; Becerra et al., 2016).

The ACC and orbitofrontal cortex have been shown to be functionally connected and involved in emotional processing, as well as having interlinked downstream output pathways to thalamus and amygdala (Garcia-Cabezas and Barbas, 2017). Disrupted thalamocortical connections to the ACC and prefrontal cortex through ischemic damage can lead to a dysexecutive syndrome (Serra et al., 2014), suggesting that functional integrity of these thalamocortical pathways is required for emotional processing and executive function. Both the hypothalamus and thalamus have limbic connections to the ACC (Morgane et al., 2005), and these regions may also be implicated in aversion to negative sensory stimuli in migraine, as part of a hypersensitive corticolimbic network (Wilcox et al., 2016).

These studies into the ACC and other frontal cortical areas in migraine and in other pain states suggest the role of these regions in the emotional processing of pain, as well as their roles in other behavioral and cognitive modalities.

### Thalamus

While classically the thalamus is well-recognized as part of the pain network within the brain and altered thalamocortical activity prior to pain onset in migraine has been shown and is likely involved in mediating early altered sensory processing (Maniyar et al., 2014; Karsan et al., 2020a). Its early role is unsurprising, given it has bidirectional projections to areas including limbic and cortical sensory areas.

The involvement of the thalamus and its cortical connections have been thought to mediate the hyperexcitability of the migraine brain to sensory stimulation and the sensitivities of the migraine brain to homeostatic alterations (Noseda et al., 2014, 2017). Functional resting state connectivity studies have demonstrated altered thalamocortical connectivity interictally in migraine and hypothesized that these changes may be involved in the mood and cognitive symptoms migraineurs can experience particularly with increasing attack frequency (Coppola et al., 2016a,b), the interictal sensitivity to homeostatic alterations and in mediating the threshold to pain through regulation of inhibition or facilitation of pain (Wang et al., 2016), as well as the role of the thalamus in chronification in migraine (Coppola et al., 2013).

We have previously demonstrated reduced functional connectivity between the thalamus and cuneus/precuneus region during the premonitory phase of migraine (Karsan et al., 2020a). The precuneus is a cortical parietal region involved in a wide spectrum of activities, including memory, visuospatial imagery, and self-consciousness (Cavanna and Trimble, 2006). The precuneus is also part of the DMN, and altered connectivity in this network has been demonstrated in migraine, predominantly interictally, with regard to decreased connectivity between the precuneus and areas outside the DMN, namely, other cortical pain-processing areas (Zhang et al., 2016). Wang et al. (2016) also used thalamic seeds to examine resting state functional connectivity between the thalami and other brain regions in interictal migraine without aura and found reduced connectivity between the posterior thalamus and precuneus/PCC region.

The cuneus is dorsal to the precuneus and is involved in primary visual processing. The cuneus and other regions of the occipital lobe (such as the lingual gyrus) have been implicated structurally and functionally in migraine, particularly in those with aura (Palm-Meinders et al., 2017). Despite its predominant role in vision, the cuneus has been implicated as playing a role in psychiatric disease such as depression (Zhong et al., 2017) and dementia with Lewy bodies (Minoshima et al., 2002); these studies thereby suggest its likely role in multisensory integration and cognitive processing.

### Basal Ganglia

Of particular recent interest in migraine is the highly dopaminergic ventral tegmental area (VTA). The substantia nigra is a dense dopaminergic nucleus in the ventral tegmentum and has emerged in a few migraine imaging studies as a potential area of interest in migraine (Welch et al., 1998; Cao et al., 2002), in particular prior to the onset of pain in the premonitory phase (Maniyar et al., 2014).

Three main dopaminergic brain pathways evolve from the VTA and substantia nigra: the mesocortical (cognitive control, motivation, and emotional response), mesolimbic (motivation, desire, reinforcement, learning, and fear), and nigrostriatal (reward, memory consolidation, and direct and indirect motor pathways) pathways. These pathways all arise from the substantia nigra and VTA areas and project to other brain areas of interest in migraine, via subcortical and cortical connections using dopamine, glutamate, and γ-aminobutyric acid (Bannon and Roth, 1983; Bertolucci-D’Angio et al., 1990; Haber et al., 2000).

The dorsal raphe nucleus (DRN) is serotoninergic and also located in the midbrain tegmentum region (Hornung, 2003). It receives afferents from several brain regions and sends projections to various other brain regions, including the caudate, putamen, and substantia nigra, as well as to the trigeminal nucleus caudalis (Imai et al., 1986) and to medial prefrontal cortex (Stratford and Wirtshafter, 1990). As well as its roles in pain (Wang and Nakai, 1994), the DRN is also involved in sleep–wake regulation via its connections to locus coeruleus and hypothalamus (Monti, 2010a,b). In addition, the DRN has been demonstrated to have a role in depression; the dorsomedial part of this nuclei group is innervated by other brain structures that can regulate mood states (Lowry et al., 2008). This nuclei group and its dopaminergic and serotoninergic projections are therefore of interest in migraine, especially as this region could be involved in the effective therapeutic response of migraine to dihydroergotamine and triptans (Vila-Pueyo et al., 2018).

In addition to the midbrain and substantia nigra, several pain imaging studies have also previously implicated other regions in the basal ganglia in pain processing and indeed in migraine (Kobari et al., 1989; Welch et al., 1998; Maleki et al., 2011; Yuan et al., 2013; Shokouhi et al., 2016). The basal ganglia circuitry with the thalamus and cortex has been implicated in the integration of motor, cognitive, emotional, and autonomic responses to pain (Chudler and Dong, 1995; Borsook et al., 2010). Additional evidence for basal ganglia involvement in migraine comes from a study demonstrating neuronal activity alterations in the caudate following cortical spreading depression, which may contribute to pain as well as other alterations in cognition and behavior in migraine (Seghatoleslam et al., 2014).

## Altered Brain Activity in Migraine and Relationship to Chronicity

Common structural areas and functional pathways between brain areas in migraine and mood and fatigue states, as well as aminergic neurotransmitter systems involving dopamine, serotonin, and noradrenaline, are therefore likely involved in the association between migraine and mood and fatigue states.

Emerging functional imaging work has suggested that longstanding and chronic disease in migraine can alter brain network function (Aurora et al., 2007; Coppola et al., 2013, 2017; Goadsby, 2013; Schwedt et al., 2013; Hubbard et al., 2014; Androulakis et al., 2017a,b; Schulte et al., 2017), and it is clear that these networks can be modulated by various means including medication overuse (Fumal et al., 2006; Grazzi et al., 2010; Riederer et al., 2013; Lai et al., 2016). Pain is a complex experience, and migraine clearly involves altered function in brain areas both within and external to regions of the pain network. Inherent to migraine as a disorder is the theory of thresholding and disordered sensory processing. Such brain alterations may be dynamic and therefore also susceptible to exogenous (Panconesi et al., 2012; Ashina et al., 2017) or endogenous (Pakalnis, 2016) triggering. Brain alterations may also become fixed with time and may be a cause or an effect of disease chronification. Chronic migraine studies have suggested fixed changes in brain structure with increased disease burden (Aurora et al., 2007; Valfre et al., 2008; Coppola et al., 2013, 2017; Goadsby, 2013; Hubbard et al., 2014; Schulte et al., 2017).

## Summary

Through review of the current and evolving literature, we have here summarized the possible links between migraine as a neural disorder, and disorders of fatigue and mood. We hypothesize that shared biological mechanisms via brain regions and neurotransmitter pathways are likely involved in the bidirectional links between migraine and these disorders, rather than true comorbidity due to other reasons. Treating underlying migraine in those with mood and fatigue complaints is likely to form an important aspect to their management. Further understanding of these diseases’ associations can be developed through increased attention to clinical trial design and prospective symptom recording by patients, as well as systematic documentation of therapeutic responses of non-painful symptoms to migraine treatments.

## Further Work

Systematic and objective quantification of non-painful disability associated with migraine will help us better understand how to classify this symptomatology in migraine and who and how we could best manage these symptoms for the benefit of patients. This increased understanding of dynamic and oscillating brain changes throughout a migraine attack, as well as more fixed functional and structural changes related to disease activity and chronicity, suggest that we now have a plausible anatomical, biological, and neurochemical link between migraine and disorders of mood and fatigue. Emerging evidence suggests that the strong implication of limbic connectivity in migraine is a feature throughout the attack (O’Carroll, 2007; Burstein and Jakubowski, 2009; Stankewitz and May, 2011; Hadjikhani et al., 2013; Wilcox et al., 2016; Chen et al., 2017; Chong et al., 2017; Karsan et al., 2020a).

This work provides a novel avenue to think about the associated mood and cognitive symptomatology in migraine and to consider objectively measuring migraine-associated disability in clinical trials, in the clinic and for research purposes, with validated measures of mood, fatigue, and cognition, so that the association of these conditions with migraine can be systematically studied in a randomized and controlled way, and associated with headache burden. In particular, given that emerging evidence suggests that effective migraine treatment could improve scores on validated measures of fatigue and cognition, incorporation of these factors involved in non-headache disability into clinical trial capture is important (VanderPluym et al., 2018). Such strategies going forward would help explore the relationship between migraine and depression or fatigue, or lead to the acceptance of depression and fatigue as accepted features of the disorder, not necessarily warranting separate treatment but being managed as part of the disorder itself.

## Conclusion

The understanding of what a migraineur actually experiences and the effective management of this are vital to the physician–patient alliance in headache medicine and in communicating disease and attack-related disability to family, friends, colleagues, employers, and schools. Many sufferers feel that their disease burden is underrecognized, ill managed, and largely attributed to psychological disease and therefore mismanaged. We hypothesize based on the current evidence that perhaps for classification, diagnosis, management, and clinical trial design, migraine should be considered a neural disorder of mainly aminergic brain function, in which alterations in networks integrating the limbic system with the sensory and homeostatic systems occur early via the thalamus, and the involvement of the pain system is one part of the process but not invariable. These networks are likely also implicated in the disorders of mood and fatigue, but in migraine have an additional role in mediating sensory hypersensitivity and pain. Systematic and objective quantification of non-painful disability associated with migraine will help us better understand how to classify this symptomatology in migraine and who and how we could best manage these symptoms for the benefit of patients. In addition, systematic data capture of non-painful symptoms in migraine and their associated disability in clinical trials going forward, and the effects of treatment, will allow evaluation of therapeutic effects on these symptoms.

## Author Contributions

NK was responsible for reviewing the literature, collating the data, and writing the manuscript. PG was responsible for reviewing the manuscript and giving expert input prior to submission. Both authors contributed to the article and approved the submitted version.

## Conflict of Interest

The authors declare that the research was conducted in the absence of any commercial or financial relationships that could be construed as a potential conflict of interest.
